# Preparation, Characterization and Performances of Powdered Polycarboxylate Superplasticizer with Bulk Polymerization

**DOI:** 10.3390/ma7096169

**Published:** 2014-08-29

**Authors:** Xiao Liu, Ziming Wang, Yunsheng Zheng, Suping Cui, Mingzhang Lan, Huiqun Li, Jie Zhu, Xu Liang

**Affiliations:** 1 College of Materials Science and Engineering, Beijing University of Technology, Beijing 100124, China; E-Mails: wangziming@bjut.edu.cn (Z.W.); cuisuping@bjut.edu.cn (S.C.); lanmingzhang@bjut.edu.cn (M.L.); huiqunli@bjut.edu.cn (H.L.); zhujie@bjut.edu.cn (J.Z.); liangxu@bjut.edu.cn (X.L.); 2 State Key Laboratory of Solid Waste Reuse for Building Materials, Beijing 100041, China; E-Mail: zhengyunsheng@outlook.com; 3 Technology Department, China Building Materials Academy, Beijing 100024, China

**Keywords:** polycarboxylate, superplasticizer, powdered, fluidity, cement

## Abstract

A polycarboxylate superplasticizer (PCE) was synthesized in a non-solvent system with bulk polymerization and then was pulverized into powdered form to achieve a rapid transportation and convenient preparation. PCE synthesized by using isopentenyl polyethylene glycol (TPEG) or isobutenyl polyethylene glycol (IPEG) as a macromonomer exhibited the best fluidities and retaining properties at 80 °C and 75 °C, respectively. Besides, azobisisobutyronitrile (AIBN) was suitable as an initiator, and the fumaric acid was suitable as the third monomer. The test results of ^1^H nuclear magnetic resonance (^1^H NMR) confirmed the occurrences of polymerization, and the measurement results of molecular weight and distribution showed that PCE molecular weight characteristics were in accordance with their fluidity properties in cement paste. The application performances in cement showed that PCEs with the best paste fluidity retentions had the longest final setting time and the shortest setting time interval, and the PCEs with good fluidity properties can obviously delay the hydration process and lower the hydration heat. Accordingly, this is a novel, energy-saving and economical method to prepare powdered PCE in the field of concrete admixtures.

## 1. Introduction

In recent years, the research and production of concrete admixtures are developing rapidly with a tendency to prepare high-performance and non-polluting concrete [1,2,3]. Polycarboxylate superplasticizer (PCE) is used for preparing high-strength, ultra-high-strength, high-flowing and self-compacting concrete for its advantages of a high water-reducing rate and good slump retention, and it is becoming a focus in the research of concrete admixtures [4,5,6,7,8,9]. In 1981, PCE had been studied in Japan, by Nippon Shokubai Co., Ltd., who initially reported a successful preparation of PCE with a certain percentage of hydrophilic functional groups [10]. From 1990 to the present, Shokubai had applied for more than 50 U.S. patents in PCE synthesis, but they were generally carried out in the solvent system. In the market today, most of the PCE products are in the form of liquid, because of the use of solution polymerization. Considering the inconvenience of liquid products’ transportation, application, storage volume [11] and concentration change, as well as the solid melamine superplasticizer generally as a main ingredient in ready-mixed mortar, some researchers had begun their studies on solid powder PCE products [12] ever since PCE was developed successfully, but most of them are still at the starting stage.

Since 2006, Plank synthesized α-allyl-ω-methoxy polyethylene glycol (APEG)-maleic anhydride superplasticizers by bulk polymerization to investigate the impact of molecular structure on their zeta potentials, adsorption behavior and dispersion effectiveness [13,14]. In 2007, patent US7265191 [15] reported a type of PCE synthesized by a three-step process. At the first step, in the presence of an initiator, the free radical copolymerization between maleic anhydride and allyl ether monomer at a certain ratio was carried out in a non-aqueous system. In 2012, patent US8227559 [16] reported a PCE synthesized by methoxy polyethylene glycol (MPEG), maleic anhydride and sodium acetate in a non-aqueous system with bulk polymerization. However, their research emphasis was not the powdered isobutenyl polyethylene glycol (IPEG) or isopentenyl polyethylene glycol (TPEG)-type of PCEs. In patent CN200780034081.5 [17], published in 2012, TOHO Chemical Industry Co., Ltd., reported the synthesis of PCE containing polyamide polyamine, but there was a small amount of water in the final product, due to the ammonium persulfate solution used in the stage of polymerization. Gui [18] reported a powder PCE prepared from liquid PCE by a centrifugal spray drying process, but the high drying temperature can bring the risks of PCE combustion and degradation.

Preparing solvent-free and powdered PCE will become a development trend for admixture research in the future, and undoubtedly, using bulk polymerization to obtain the solid PCE product is an efficient way to achieve pulverization, because of its advantages, such as a pure product without any other solvent or reaction medium, a simple production process and low equipment investment and production cost. Therefore, in view of the cost savings and energy conservation, it is necessary to investigate PCE bulk polymerization technology, so as to prepare powdered PCE.

In this study, PCE was synthesized with bulk polymerization and then was pulverized into powder. The influences of the initiator, macromonomer, reaction temperature and third monomer on cement paste fluidities are described. PCE structures and molecular weights are characterized by ^1^H nuclear magnetic resonance (^1^H NMR) and gel permeation chromatography (GPC), respectively. Additionally, the setting times and hydration heats of cements mixed with PCEs are measured and discussed. The conveniences of loading, unloading, space saving and being prepared at an arbitrary concentration for the powdered PCE can widen the application of solid PCE in construction engineering.

## 2. Results and Discussion

### 2.1. Effects of Initiator, Macromonomer and Reaction Temperature on the Fluidities of Cement Paste

**Figure 1:**
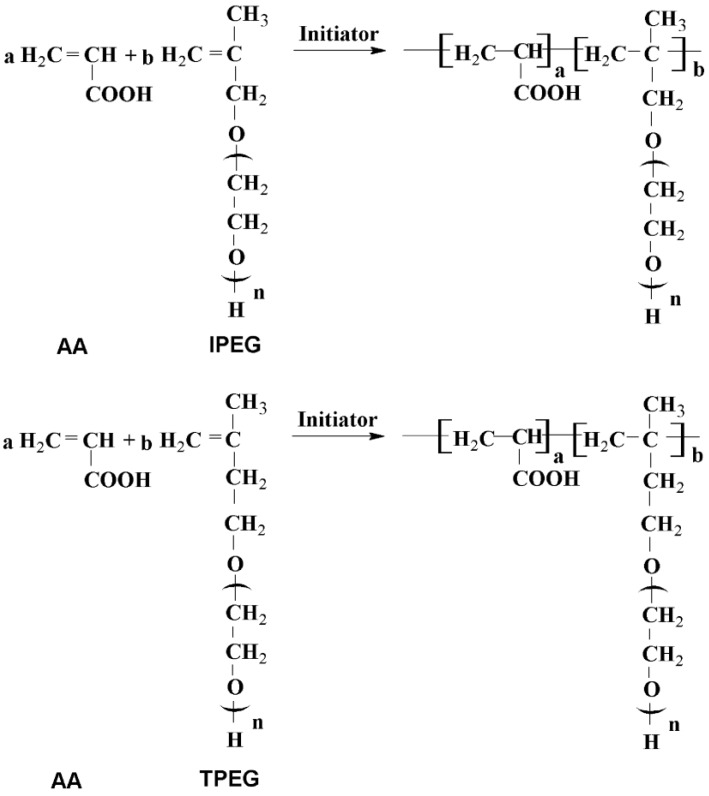
Schematic diagram of the synthesis of polycarboxylate superplasticizer (PCE). IPEG, isobutenylpolyethylene glycol; TPEG, isopentenyl polyethylene glycol.

The schematic diagram of the PCE synthesis by acrylic acid (AA) and macromonomer is shown in Figure 1. The chemical and mineral compositions of reference cement determined by Bogue calculation are illustrated in Table 1.

**Table 1:** Chemical and mineral compositions of the reference cement.

**Composition **	**SiO_2_/%**	**CaO/%**	**Al_2_O_3_/%**	**Fe_2_O_3_/%**	**MgO/%**	**SO_3_/%**	**Na_2_O/%**
Reference cement	22.93	66.23	4.29	2.89	1.92	0.35	0.70
**Composition **	**C_3_S/%**	**C_2_S/%**	**C_3_A/%**	**C_4_AF/%**	**Loss/%**	**f-CaO/%**	–
Reference cement	58.78	21.38	6.49	8.77	1.48	0.64	

Two thermal initiators involving benzoyl peroxide (BPO) and azobisisobutyronitrile (AIBN) were chosen as the initiators used for polymerization, as well as IPEG and TPEG as the macromonomers. The half-life period of BPO is 10 h at 73 °C, and BPO can dissolve in liquid IPEG or TPEG above 75 °C. The half-life period of AIBN is 10 h at 64 °C, and AIBN also can dissolve in liquid IPEG or TPEG above 75 °C. Therefore, by using BPO as the initiator, the polymerization temperature was set at 80 °C, 85 °C and 90 °C to be investigated, respectively. Similarly, when AIBN was used as the initiator, the polymerization temperature was set at 75 °C, 80 °C and 85 °C, respectively. The polymerization temperature should not be set at a higher temperature to avoid the risk of a side reaction and polyethylene glycol’s decomposition. The fluidities of cement pastes for PCEs synthesized at these different conditions are shown in Table 2. All of the used PCE dosages were 0.2% bwoc (by weight of cement).

**Table 2:** Fluidities of cement pastes for PCEs synthesized at different conditions (PCE samples: purified; PCE dosage: 0.2% bwoc (by weight of cement)). BPO, benzoyl peroxide; AIBN, azobisisobutyronitrile.

Initiator	Macromonomer	Temperature/°C	Fluidity/mm		
			5 min	1 h	2 h
BPO	IPEG	80	–	–	–
	TPEG	80	–	–	–
	IPEG	85	210 ± 5	80 ± 10	–
	TPEG	85	–	–	–
	IPEG	90	–	–	–
	TPEG	90	–	–	–
AIBN	IPEG	75	315 ± 5	180 ± 5	165 ± 0
	TPEG	75	247.5 ± 2.5	160 ± 5	100 ± 10
	IPEG	80	295 ± 0	130 ± 10	120 ± 5
	TPEG	80	260 ± 0	255 ± 5	250 ± 0
	IPEG	85	187.5 ± 2.5	85 ± 10	–
	TPEG	85	197.5 ± 2.5	190 ± 5	147.5 ± 2.5

As for BPO as the initiator in Table 2, there was no fluidity value of cement paste for PCEs synthesized at 80 °C and 90 °C. Only cement paste for PCE synthesized at 85 °C had an initial fluidity value of 210 mm, but almost lost fluidity entirely after an hour, indicating poor fluidity retention. Overall, PCE with good cement paste fluidity and fluidity retention cannot be synthesized by using BPO as the initiator for the system studied here. This is possible mismatch between BPO and the monomers used for polymerization. Although BPO can dissolve in a liquid macromonomer at high temperature, the reactivity difference of free radicals might lead to reduction of the polymerization conversion.

As for AIBN as the initiator and IPEG as the macromonomer in Table 2, cement paste for PCE synthesized at 75 °C exhibited more excellent initial fluidity and fluidity retention than that for PCE synthesized at 80 °C, indicating a better reactivity match between IPEG and AA at 75 °C than 80 °C. Cement paste for PCE synthesized at 85 °C exhibited the worst initial fluidity and fluidity retention, and even no fluidity after 2 h, which indicates the low efficiency of polymerization between IPEG and AA by the initiation of AIBN at 85 °C. This may be due to coupling termination of too many free radicals decomposed by AIBN, but not chain growth. Therefore, it is suitable for an AIBN-IPEG system to set the polymerization temperature at 75 °C.

Furthermore, from Table 2, for the AIBN-TPEG system, cement paste for PCE synthesized at 80 °C distinctly exhibited the most excellent initial fluidity and fluidity retention. Compared with cement paste for PCE synthesized at 75 °C, cement paste for PCE synthesized at 85 °C exhibited better fluidity retention, but worse initial fluidity. This is possibly because of the reactivity mismatch between the macromonomer and AA at this unsuitable reaction temperature. As a result, a certain amount of TPEG cannot react completely, leading to low copolymerization efficiency and reduced effective components in the product. Therefore, it is suitable for an AIBN-TPEG system to set the polymerization temperature at 80 °C.

In summary, AIBN as the initiator is more suitable than BPO as the initiator to synthesize PCE in a non-aqueous condition with bulk polymerization for the IPEG/TPEG system, though good results can be obtained by using BPO as the initiator for an APEG system [13]. Furthermore, each macromonomer has an optimum temperature matched with different initiators. Too high or too low of a temperature can lead to a reactivity mismatch, which decreases the polymerization efficiency and the effective components in the polymerization product. Typically, a good fluidity of cement paste can be achieved when PCE is synthesized at 75 °C by a macromonomer of IPEG or at 80 °C by a macromonomer of TPEG. This is because the p-π conjugated effect of IPEG, which has one –CH_2_ less than TPEG, leads to thicker electron cloud density and higher reactivity, showing a lower polymerization temperature (75 °C). Similarly, a relatively higher polymerization temperature (80 °C) for TPEG is necessary. From their paste fluidities, good initial fluidities and fluidity retentions of cement pastes for PCEs are easily obtained by using IPEG and TPEG as macromonomers, respectively.

### 2.2. Effects of Addition of the Third Monomer on the Fluidities of Cement Paste

Generally speaking, the addition of a third monomer can regulate the product’s molecular structure, involving the main chain arrangement and the side chain density, which is beneficial to achieve excellent performances. In this study, the fluidities of cement pastes for PCEs synthesized by using maleic anhydride (MA) or fumaric acid (FA) as the third monomer, AIBN as the initiator, TPEG as the macromonomer and 80 °C as the polymerization temperature were investigated. All of the used PCE dosages were 0.2% bwoc. The results and the comparative data from Table 2 for the same system without the third monomer are shown in Table 3.

**Table 3:** Fluidities of cement pastes for PCEs synthesized by adding the third monomer (PCE samples: purified; PCE dosage: 0.2% bwoc). MA, maleic anhydride; FA, fumaric acid.

Third Monomer	Fluidity/mm		
	5 min	1 h	2 h
MA	137.5 ± 2.5	–	–
FA	280 ± 5	245 ± 5	210 ± 10
None	260 ± 0	255 ± 5	250 ± 0

From Table 3, cement paste for PCE synthesized by adding MA as the third monomer exhibited low initial fluidity and even no fluidity after an hour. However, better initial fluidity and fluidity retention were exhibited when FA was chosen as the third monomer. The possible reason for the worse effect of MA than FA is that MA cannot hydrolyze in the non-aqueous system. Generally, during the synthesis of conventional PCE, MA firstly hydrolyzed to carboxylic acid and then copolymerized with other monomers. Therefore, there was a large number of residual monomers and polymerization product without an ideal structure if MA was added as the third monomer into a non-aqueous system. Additionally, the non-stretched carboxyl groups on the same side of MA with a *cis*-structure in the non-aqueous system may lead to a decline of the polymerization efficiency.

Compared with the paste fluidity data for PCE synthesized in the AIBN-TPEG system at 80 °C without the third monomer, the paste fluidity data for PCE using FA as the third monomer showed that adding FA slightly changed the corresponding paste fluidity result, including the increased initial fluidity and the decreased fluidity retention. This indicates that FA as the third monomer has good polymerization adaptability with TPEG in that system, but decreases the fluidity retention, especially after 2 h. That FA is more suitable as the third monomer than MA may be caused by two improvements: one is that FA is not an anhydride, but carboxylic acid, which can react in a non-aqueous system without hydrolysis; the other is that FA with a trans-structure more easily exhibits a stretched structure in space than MA and, thus, decreases the possibility of the reduced polymerization degree by chain entanglement.

### 2.3. ^1^H NMR of PCE

**Figure 2:**
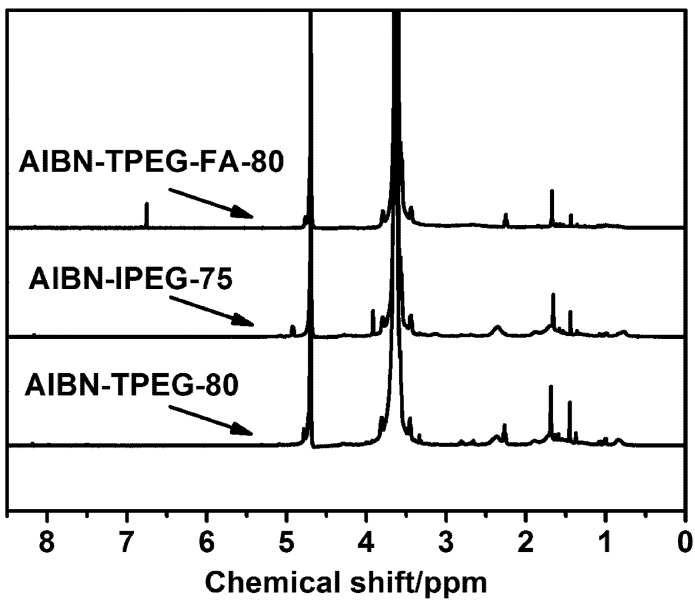
^1^H NMR spectra of PCEs (PCE samples: purified).

PCE samples synthesized in the AIBN-TPEG system at 80 °C, in the AIBN-IPEG system at 75 °C and in the AIBN-TPEG system at 80 °C with FA as the third monomer are identified as AIBN-TPEG-80, AIBN-IPEG-75 and AIBN-TPEG-FA-80, respectively. The ^1^H NMR spectra of these three samples are shown in Figure 2, and these spectra were analyzed by means of the reported references [19,20].

There are very similar spectra curves that exhibit some common features in Figure 2. Typically, in the main chain, –CH_2_, the –CH connected carboxyl group and –CH_2_ from TPEG or IPEG gave signals at δ = 1.454, 1.678 and 2.267 ppm, respectively; in the side chain, the strong peaks at 3.60 to 3.75 ppm corresponded to –CH_2_CH_2_O– in polyethylene oxide, for the side chain contained more than approximately 58 units of ethylene oxide (EO). Besides, the peaks at 4.70 ppm corresponded to D_2_O used for dissolving the sample. Comparing the spectrum of AIBN-TPEG-FA-80 with the spectra of AIBN-IPEG-75 and AIBN-TPEG-80, there appeared an observed peak at 6.752 ppm in the spectrum of AIBN-TPEG-FA-80, and this peak corresponded to the –CH from FA. Based on the above analysis, all of the sample products certainly had characteristic functional groups and, thus, showed a relatively completed copolymerization between AA and the macromonomer, including FA as the third monomer for AIBN-TPEG-FA-80.

### 2.4. Molecular Weight of PCE

**Figure 3:**
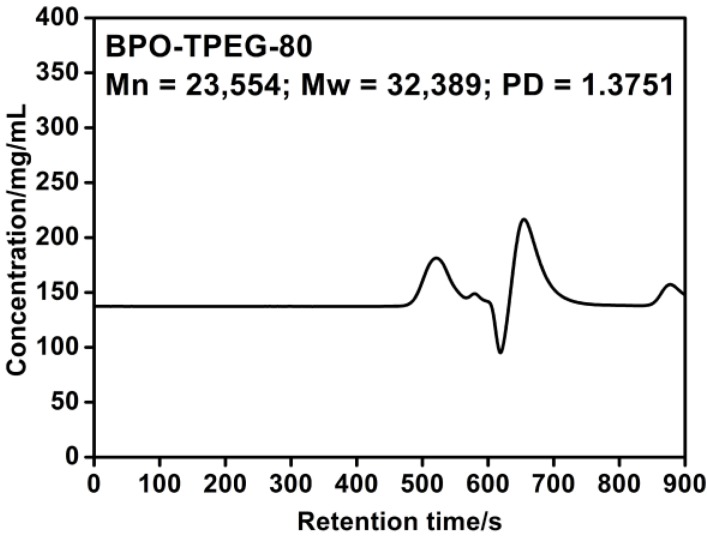
Molecular weight of PCE (initiator: BPO; PCE samples: purified).

The molecular weight results of PCEs synthesized at 80 °C with BPO as the initiator and TPEG as the macromonomer are shown in Figure 3. The peaks at the left side and at the right side corresponded to the PCE polymerization product and the unreacted monomers, respectively. There was a high peak on the right side, further proving the low initiation efficiency in the BPO initiating system, which was in accordance with its paste fluidity data.

**Figure 4:**
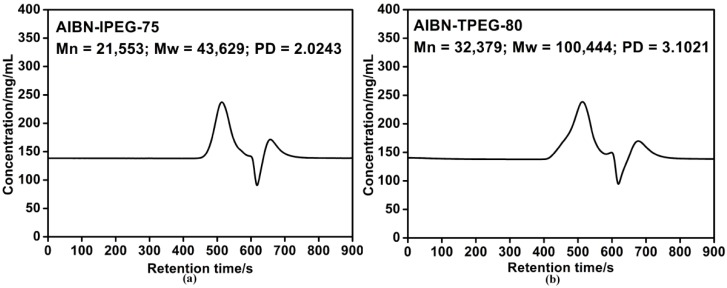
Molecular weight of PCEs: (**a**)AIBN+IPEG; (**b**) AIBN+TPEG (PCE samples: purified).

The molecular weight results of PCEs synthesized at optimum conditions with AIBN as the initiator and IPEG (Figure 4a) or TPEG (Figure 4b) as the macromonomer are shown in Figure 4. The right peaks in Figure 4a,b were both lower than those in Figure 3, indicating that most of the monomers had participated in polymerization, and thus, there were better paste fluidities for the AIBN initiating system than the BPO initiating system. The high polydispersity (PD) values in Figure 4 implied the wide molecular weight distribution of polymerization products with different polymerization degrees. In summary, products initiated by AIBN exhibited more excellent paste fluidities and fluidity retentions owing to their fewer unreacted monomers and higher initiation efficiency.

Furthermore, from Figure 3 and Figure 4, there was a slight peak with the retention time of 570–600 s between the PCE peak and the unreacted monomer peak. The peak at this position implies a molecular weight of about 10,000. According to the characteristic of self-polymerization for AA, it can be inferred that this peak likely corresponded to the homopolymerized AA in the reaction products. For the higher viscosity in the non-aqueous system, it is difficult to achieve the AA monomers’ complete copolymerization with other macromonomers without any self-polymerization. The slightly lower AA peaks were consistent with the lower monomer peaks in Figure 4 compared to Figure 3, further indicating the higher copolymerization degree and better performances for the AIBN initiating system than the BPO initiating system.

### 2.5. Setting Times of Cement Pastes

Table 4 shows the setting times of cement pastes for PCEs synthesized with bulk polymerization. The blank sample had no PCE, and the other samples’ PCE dosages were 0.2% bwoc.

**Table 4:** Setting times of cement pastes with PCEs (PCE samples: purified).

Sample	Dosage/%	Water/g	Setting Time	
			Initial Setting/min	Final Setting/min
Blank	0	138	197 ± 3	250 ± 5
AIBN-IPEG-75	0.2	115.1	197 ± 5	240 ± 2
AIBN-IPEG-80	0.2	117.4	178 ± 1	235 ± 3
AIBN-TPEG-75	0.2	116.2	160 ± 2	210 ± 5
AIBN-TPEG-80	0.2	124.2	184 ± 2	259 ± 3
AIBN-TPEG-85	0.2	122.9	201 ± 3	233 ± 3
AIBN-TPEG-FA-80	0.2	118.1	189 ± 1	225 ± 4

From Table 4, adding PCE had different influences on the setting times of cement paste. The final setting time of cement paste for AIBN-TPEG-80 was the longest, but the other pastes’ final setting times were all shorter than that of the blank sample. The possible reason is that adding PCE can reduce the formation of the flocculent structure of cement particles, manifesting as the reduction of the calcium concentration in the liquid phase during initial hydration by means of forming unstable complexes between the –COOH of PCE and calcium ions in the cement paste. Thereafter, the hydration was delayed, and a retarding effect was shown, which was in accordance with the good fluidity retention. The interval between final setting time and initial setting time of cement paste for AIBN-TPEG-85 was the shortest, probably because the unstable complexes between the –COOH of PCE and the calcium ions in the cement paste gradually decompose during the hydration process, resulting in normalized hydration and a shortened final setting time, which is related to the lower fluidity values.

### 2.6. Hydration Heat

**Figure 5:**
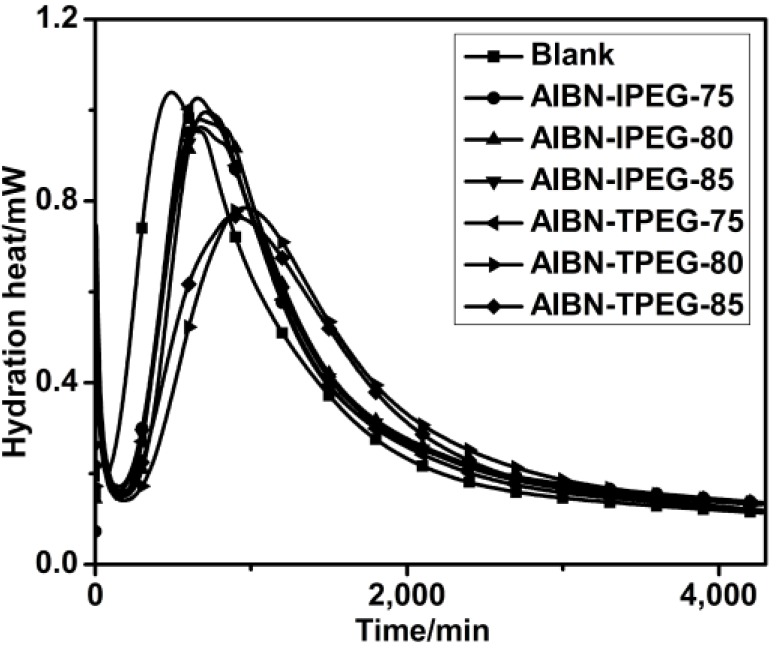
Hydration heats of cements with PCEs (PCE samples: purified).

The hydration heat and hydration rate of reference cements mixed with six self-synthesized PCEs, respectively, within three days are shown in Figure 5. The blank sample had no PCE, and the other samples’ PCE dosages were 0.2% bwoc.

From Figure 5, adding PCE can delay the hydration process and reduce the maximum heat value. Compared with the blank sample, AIBN-TPEG-80 and AIBN-TPEG-85 both exhibited a more delayed hydration process (a later hydration heat peak appeared) and lower hydration heat values. This may be related to their good paste fluidity retentions. During the induction period of hydration, adding AIBN-TPEG-80 or AIBN-TPEG-85 can prompt the formation of complexes between PCE and the calcium ions in the cement paste, leading to the reduction of the calcium concentration and inhibition of the forming of C–S–H. As a result, the induction period had to be prolonged, manifesting as the good paste fluidity retention. The other four samples all exhibited a bit longer induction period than the blank sample and nearly the same hydration heat values as that of blank sample, indicating the similar effect on the hydration process.

### 2.7. Preparation and Dispersion Mechanisms of PCE

The powdered PCE was prepared via bulk polymerization. As one of the polymerization methods, it had the outstanding feature of its procedure having no solvent. Therefore, this polymerization method was used for synthesizing solid-state PCE directly. By this means, it can save energy and manpower to separate the solvent in the final products, which was easy to do in powdered form. In this paper, the polymerization mechanism is the free radical polymerization among acrylic acid and macromonomers. From what has been discussed above, powdered PCE with good performances is prepared successfully, and the possible difficulties of high viscosity and poorly released heat are overcome. A novel way to simply and economically prepare powered PCE is provided.

The dispersion mechanism of PCE was discussed based on the current theories and measured structure-property results. Several types of non-contact interactions occurred within a cementitious suspension [21]. The mechanism for PCE in a cement-water system mainly included electrostatic repulsion, steric hindrance and lubrication [22,23,24].

As for electrostatic repulsion, cement particles interacted via (generally attractive) van der Waals forces [25] at a short distance; besides, there were electrostatic forces that resulted from the presence of adsorbed ions at the surface of the particles [26]. When PCE adsorbed on the surfaces of cement particles, according to the theory of PCE electrostatic repulsion [27,28], the electrostatic repulsion occurred among the identical charged cement particles caused by carboxylate groups, leading to the stable suspension and good dispersion for the cement particles in the cement-water system.

As for steric hindrance, which was believed to predominate over the electrostatic repulsion, the PCE main chains adsorbed on the surfaces of cement particles, and their side chains stretched in aqueous phase to form a thick adsorbed layer. Then, the cement particles repelled each other, and a good dispersion was achieved by this steric hindrance effect [29].

As for lubrication, according to Byung-Gi Kim’s research [30], a water film with a certain strength formed on the surfaces of particles can break the flocculated cement particles and improve the flow properties. Thus, the cement particles can be dispersed homogeneously by the synthesized powered PCE in the cement-water system.

The mechanism of powered PCE in the cement-water system is similar to that of common PCE. The improvements of the paste’s rheological properties are attributed to the added PCE adsorption. The separation distance is an important parameter to characterize the paste’s rheological properties. Each of the different interactions introduces non-contact forces between particles, the magnitude of which depends primarily on their separation distance. Repulsive electrostatic forces alone are generally insufficient to prevent agglomeration, due to van der Waals attractive forces; and steric hindrance or additional electrostatic repulsion from polymers is needed to disperse cement particles [31]. A quantitative measurement had been carried out, and the relationship between cement grains’ separation distances and the paste yield stress were reported by A. Perrot *et al.* [32]. For the non-admixed reference paste, the surface-to-surface separation distance is 1.5 nm and is very much in line with previous experimental results. At full surface coverage, when PCE is added, it is equal to two times the thickness of the adsorbed polymer layer (*i.e.*, 5 nm). Without any polymers, it is equal to two times an equivalent layer thickness at zero surface coverage. At intermediate surface coverage, it results from the contribution of the pairs of particles that do interact without any PCE, the pairs of particles that do interact via one adsorbed layer of PCE on one of the particles and the pairs of particles that do interact via one adsorbed layer of PCE on each of the particles. The increased separation distance between cement particles in the PCE-added paste can improve its rheological characteristic, which can bring a link between macroscopic rheology and the microscopic adsorption of polymers.

## 3. Experimental Section

### 3.1. Materials

The isobutenyl polyethylene glycol (IPEG) and isopentenyl polyethylene glycol (TPEG), both with molecular weights of 2,400, were from Liaoning Kelong Fine Chemical Co., Ltd. (Liaoyang, China). The acrylic acid (AA), maleic anhydride (MA), benzoyl peroxide (BPO) and azobisisobutyronitrile (AIBN) were all purchased from Tianjin Fuchen Chemical Reagents Factory (Tianjin, China). The fumaric acid (FA) and mercaptoacetic acid were both supplied by Tianjin Guangfu Fine Chemical Research Institute (Tianjin, China). Reference cement P. I. 42.5 used for performance testing and superplasticizer analysis was supplied by China United Cement Qufu Co., Ltd. (Qufu, China).

### 3.2. Synthesis of PCE

The macromonomer (IPEG or TPEG, 0.03 mol) was added to a 500-mL, four-neck, round-bottomed flask with a stirrer, a thermometer and an intelligent control temperature device, and then, the temperature was slowly raised to the fixed temperature by stirring and heating. When the macromonomer was completely melted, AA (0.12 mol, the amount was changed to 0.015 mol if there was a third monomer), the third monomer (0.06 mol, if any), mercaptoacetic acid (0.006 mol) and the initiator (0.005 mol) were added in order, and this temperature was maintained constant for reacting for 6 h. After the polymerization, the product was cooled to room temperature and was in solid form. Finally, the products were subjected to several cycles of repeated precipitation in an excess amount of anhydrous ethyl ether to remove any residuals (including non-reacted macromonomers and other impurities) and were dried under vacuum for one day at room temperature for further characterizations and measurements.

### 3.3. Preparation of Powdered PCE

The solid PCE product was pulverized in a FW100 high-speed universal pulverizer (Tianjin Taisite Instrument Co., Tianjin, China) to obtain a powdered PCE. Then, it was mixed with some water to prepare a PCE solution with the required concentration. The specimens used for the further tests were purified by repeated precipitation with excess ethyl ether.

### 3.4. Tests and Measurements

#### 3.4.1. ^1^H NMR Analysis

Three dried PCE samples were purified via dissolution and precipitation at least three times and then dried in a vacuum at 60 °C for 24 h to a constant weight. The ^1^H NMR spectrum was obtained at room temperature (25 °C) with an ARX-400 spectrometer (Bruker Co., Rheinstetten, Germany) operating at a frequency of 400 MHz, and the chemical shift values were expressed in δ values (ppm) relative to tetramethylsilane (TMS) as the internal standard. A sample for ^1^H NMR was prepared by dissolving in the solvent, *i.e.*, deuterated water (D_2_O), as the internal reference.

#### 3.4.2. Molecular Weight and Its Distribution

The number average molecular weight (Mn), weight average molecular weight (Mw) and polydispersity index (PD) of three PCEs were determined at 30 °C on a PL-GPC50 (Polymer Laboratories, Church Stretton, UK) equipped with a PL aquaqel-OH MIXED 8-µm column and three detectors, including a differential refraction detector, a laser light scattering detector and a viscosity detector. The molecular weight results can be measured directly without standard sample calibration [33]. The calculation of molecular weight needed the refractory incremental (*dn*/*dc*) value, which was automatically calculated through the inputted sample concentration value and the detected refractive index value by the differential refraction detector. The mobile phase was water as a solvent, with an injection volume of 100 mL and a flow rate of 1 mL/min.

#### 3.4.3. Fluidity of Cement Paste

The fluidities of fourteen fresh cement pastes with PCEs were tested according to the standard method GB/T8077-2000 [34], described in the National Standards of the People’s Republic of China. The fluidity of cement paste over time was measured using the standard method. Generally, the PCE dosage was 0.2 wt% of cement, and the water-cement ratio (W/C) was 0.29 for the study if there were no particular indication. To investigate the fluidity retention, cement pastes mixed with PCEs were examined every 60 min within a total period of 120 min.

For each test of cement paste fluidity, the cement paste mixture was poured into a truncated cone (60 mm height, 36 mm top diameter and 60 mm bottom diameter) on a glass plate, and then, the cone was vertically removed. After 30 s, the diameter of the paste was recorded as the fluidity of the paste, and the resulting spread of the paste was measured twice. The second measurement was perpendicular to the first measurement, and the average was calculated to yield the spread value.

#### 3.4.4. Setting Times of Cement Paste

The setting times of seven fresh cement pastes with or without PCE were tested according to the standard method, GB/T50080-2002 [35], described in the National Standards of the People’s Republic of China. Each sample was tested twice, and the average was calculated as the setting time value.

#### 3.4.5. Hydration Heat

The hydration heat and hydration exothermic rate for seven cement pastes with or without PCE were measured by using a TAM AIR-08 Thermostat (Thermometric, Järfälla, Sweden), according to the reported method [36]. The mass of cement sample to be tested was 3 g with a W/C of 0.5, and the dosage of PCE was 0.2 wt% of cement.

## 4. Conclusions

Powdered PCE can be successfully prepared in a non-aqueous condition with bulk polymerization followed by pulverization. PCE synthesized by AIBN as the initiator exhibited better paste fluidity results than PCE synthesized by BPO as the initiator.

The good initial fluidity of the cement paste can be obtained when PCE was synthesized at 75 °C by a macromonomer of IPEG or when PCE was synthesized at 80 °C by a macromonomer of TPEG, as well as with FA as the third monomer.

GPC measurements showed that the products initiated by AIBN exhibited fewer unreacted monomers and higher initiation efficiency, manifesting as better paste fluidity performances. ^1^H NMR spectra confirmed the characteristic functional groups of PCEs.

AIBN-TPEG-80 and AIBN-TPEG-85 exhibited the longest final setting time and the shortest setting time interval of cement paste, respectively. Their more delayed hydration process and lower hydration heat were consistent with their good paste fluidity retentions.

## Author Contrbutions

Xiao Liu wrote and submitted the manuscript. He also contributed to searching the related other researcher’s literatures and achievements, results analysis, experiment design and doing all of synthetic experiments; Ziming Wang revised the manuscript and provided the research ideas; Yunsheng Zheng, Suping Cui, Mingzhang Lan, Huiqun Li, Jie Zhu and Xu Liang assisted with ^1^H NMR measurements, GPC measurements, hydration heat measurements, PCE pulverization, fluidity performance testing and setting time testing, respectively.
